# Spatial and environmental effects on Coho Salmon life history trait variation

**DOI:** 10.1002/ece3.6912

**Published:** 2020-10-17

**Authors:** Kimberly M. F. Tuor, Daniel D. Heath, J. Mark Shrimpton

**Affiliations:** ^1^ Fisheries Protection Program, Fisheries and Oceans Canada Whitehorse Yukon Canada; ^2^ Great Lakes Institute for Environmental Research and Department of Integrative Biology University of Windsor Windsor ON Canada; ^3^ Ecosystem Science & Management (Biology) Program University of Northern British Columbia Prince George BC Canada

**Keywords:** adaptation, environment, migration, reproductive investment, trade‐offs

## Abstract

Adult size, egg size, fecundity, and mass of gonads are affected by trade‐offs between reproductive investment and environmental conditions shaping the evolution of life history traits among populations for widely distributed species. Coho salmon *Oncorhynchus kisutch* have a large geographic distribution, and different environmental conditions are experienced by populations throughout their range. We examined the effect of environmental variables on female size, egg size, fecundity, and reproductive investment of populations of Coho Salmon from across British Columbia using an information theoretic approach. Female size increased with latitude and decreased with migration distance from the ocean to spawning locations. Egg size was lowest for intermediate intragravel temperature during incubation, decreased with migration distance, but increased in rivers below lakes. Fecundity increased with latitude, warmer temperature during the spawning period, and river size, but decreased in rivers below lakes compared with rivers with tributary sources. Relative gonad size increased with latitude and decreased with migration distance. Latitude of spawning grounds, migratory distance, and temperatures experienced by a population, but also hydrologic features—river size and headwater source—are influential in shaping patterns of reproductive investment, particularly egg size. Although, relative gonad size varied with latitude and migration distance, how gonadal mass was partitioned gives insight into the trade‐off between egg size and fecundity. The lack of an effect of latitude on egg size suggests that local optima for egg size related to intragravel temperature may drive the variation in fecundity observed among years.

## Graphical abstract

We examined the effect of environmental variables on female size, egg size, fecundity, and reproductive investment for populations of Coho Salmon from across British Columbia. Our work is novel because we combined location variables (latitude and migration distance) with environmental variables (water temperature, river size, headwater source, and years of hatchery supplementation). We found that latitude of spawning grounds, migratory distance, and temperatures experienced by a population, but also hydrologic features—river size and headwater source—are influential in shaping patterns of reproductive investment, particularly egg size.

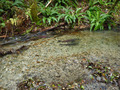

## 1 INTRODUCTION

Understanding how individual organisms successfully exploit habitats within their range is important to fully understand local adaptation which in turn impacts management efforts for the species, not only at the population level, but throughout their geographic range. This is especially important when working with and managing species of concern because conservation efforts would benefit from a fuller understanding of the species' physiology, ecology, and life history. A species able to exploit a range of habitats throughout their distribution may use different life history traits and display different trade‐offs to maximize fitness (Lappalainen et al., 2008). Specific differences in life history traits among populations during the spawning and incubation period are of particular interest in Pacific salmon because the highest rates of mortality occur during the incubation and alevin stages (Quinn, 2005).

Svardson (1949) suggested that allocation of resources to egg production, defined as the product of egg number and egg size, will be optimized but may vary due to local selection pressures. There is, however, a trade‐off between the size and number of eggs produced, which precludes investing maximally in both traits simultaneously (Svardson, 1949). The number of eggs, therefore, will vary in response to selective pressures both on egg size and total investment in egg production (Fleming & Gross, 1990). Previous work has shown that absolute fecundity of anadromous salmon generally increases with latitude (Beacham, 1982; Healey & Heard, 1984; Fleming & Gross, 1990; but see Rounsefell, 1957; McGurk, 2000). Fecundity also increases with female size (Campbell et al., 2006; Leggett & Carscadden, 1978; Rounsefell, 1957). The increase in fecundity with latitude, therefore, could be attributed to larger body size of female Pacific salmon from more northerly populations. Beacham and Murray (1993), however, found that the increase in fecundity with latitude was independent of body size for all Pacific salmon, except Chum Salmon (*Oncorhynchus keta*). Given the trade‐off between egg size and number, a decrease in egg size at higher latitude would be predicted for Pacific salmon. Earlier work by Beacham and Murray (1993) found that for Pacific salmon, egg size was smaller for more northerly populations, except for Pink Salmon (*O. gorbuscha*).

Life history theory predicts that egg number will evolve around selection for egg size and Fleming and Gross (1990) suggest that local optima in egg size may drive the latitudinal clines observed in egg number for Pacific salmon. Smaller eggs of Pacific salmon from higher latitudes, however, does not necessarily result in smaller fry at emergence due to the greater metabolic efficiency at cooler temperatures, limiting the penalty of producing small eggs on fry size at emergence (Murray et al., 1990)—but few studies have measured intragravel temperature directly. Surface water temperatures have been recorded for many systems, but may be a poor surrogate for intragravel temperatures that are experienced by embryo and larval salmon (Tuor & Shrimpton, 2019). Maternal resource allocation may be further constrained by energetic cost of migration to spawning locations. Chinook Salmon (*O. tshawytscha*) from interior populations that make long upstream migrations had smaller gonads relative to somatic size and smaller eggs than coastal populations with short upstream migrations (Healey, 2001). Populations that undertake longer migrations and more energetically demanding migrations have smaller eggs because they have less energy to devote to gonadal mass and egg number is set early in migration (Braun et al., 2013; Kinnison et al., 2001). Additionally, water temperature at the time of migration is directly linked to metabolic costs of migration (Brett, 1995) and the effect on life history traits of anadromous salmon has not been evaluated.

There are also a number of other environmental factors for which locally adapted traits may be selected. Water temperature, photoperiod, and stream morphology, such as river size, have been suggested to be among the strongest freshwater abiotic factors that exert selective pressure on local adaptation in salmon (Garcia de Leaniz et al., 2007). Redd sites are characterized by gravel size, water depth, and velocity (Sandercock, 1991), and hydrologic characteristics influenced by stream size may influence life history traits. For example, smaller eggs with greater surface‐area to volume are expected in systems with smaller gravel size due to reduced porosity and oxygen levels (Quinn et al., 1995; Rollinson & Hutchings, 2011). Female spawner size determines the size and depth of redds and the largest gravel sizes suitable for use as redd substrates (MacIsaac, 2010). Additionally, rivers below lakes exhibit less sediment loading and smaller variations in discharge and daily temperature variation (Goodman et al., 2011; Milner & Bailey, 1989; Quinn, 2005). Embryo and larval survival is dependent on spawning bed quality, which is affected by sediment infiltration in the gravel and particle size distribution (MacIsaac, 2010). Increased daily variation in temperature also stresses fish, alters metabolic rate and energy use (Beauregard et al., 2013), decreases growth (Shrimpton et al., 2007) and may select for larger egg size and larger spawners.

Many Pacific salmon populations are also augmented by hatchery enhancement programs, but it has been argued that raising animals in benign environments may relax selective pressures—altering traits that are locally adapted. Heath et al. (2003) found that hatchery rearing relaxed natural selection favoring large eggs in Chinook Salmon, allowing fecundity selection to drive exceptionally fast evolution of small eggs. Earlier work by Beacham and Murray (1993), however, did not find evidence of a difference in egg size between hatchery and wild Coho Salmon suggesting no effect of hatchery supplementation.

**FIGURE 1:**
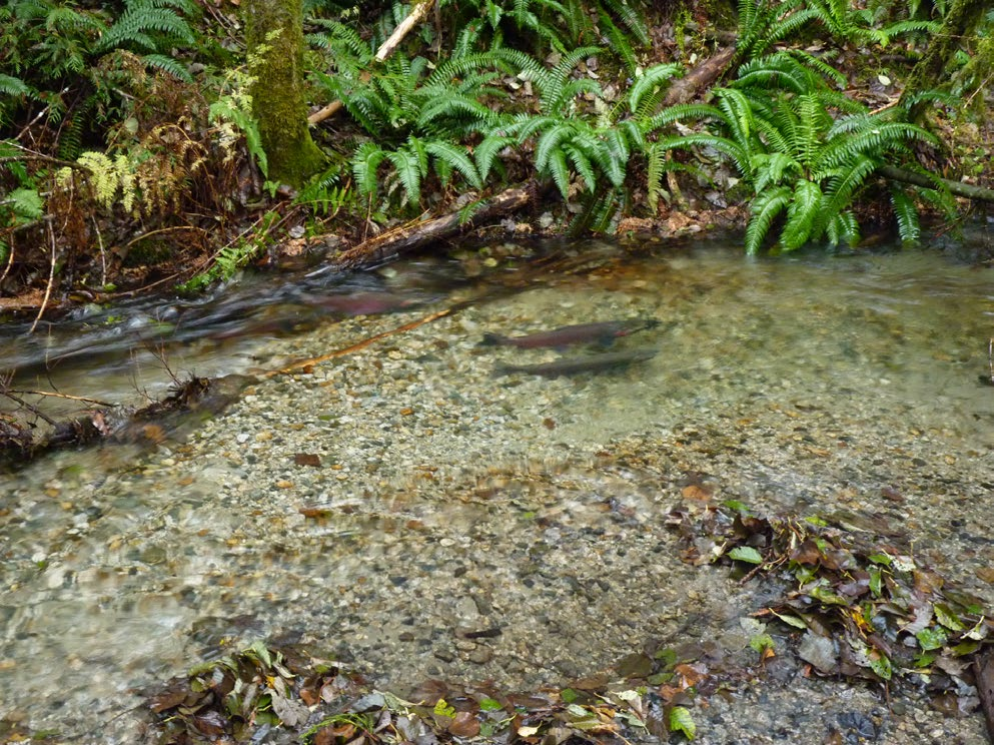
Spawning Coho Salmon in a tributary to the Bella Coola River. Photo: K.M.F. Tuor

We used Coho Salmon (*O. kisutch*) in our study (Figure 1) due to wide range of environmental variables experienced throughout their geographic distribution. Moreover, throughout British Columbia (BC) Coho Salmon spawn in over 970 rivers and small streams (Sandercock, 1991). With such a large geographic distribution, with spawning occurring along the coast as well as through the interior of BC, populations of Coho Salmon experience different ranges of temperatures during spawning and incubation and are expected to tolerate a wide range in temperatures. Variation in life history strategies among populations of Coho Salmon has been linked to both spatial and temporal effects, but rarely have studies incorporated other environmental factors that may influence life history traits. Such variables include migration distance, temperature for migrating spawners and also temperature in the redd environment, as well as river size and headwater source, both of which will influence temperature and productivity of each watershed. Our objective was to determine how life history traits differ among populations of Coho Salmon, both spatially and temporally, throughout British Columbia, and how life history trait variation relates to environmental conditions found within the incubation habitat. We used an information theoretic approach to identify a priori combinations of environmental variables that have the greatest influence on female length (*L*), egg size (*M*_egg_), fecundity (*F*), and female relative gonad size index (*I*_G_) for Coho Salmon from populations throughout British Columbia. These analyses determined factors that significantly contribute to variation in life history traits for Coho Salmon populations across their distribution in British Columbia, but also how environmental variables interact, which has not been addressed in previous studies. Characterization of life history variation across geographical and environmental gradients will help in guiding effective management and to define the nature of local adaptation in natural populations.

## 2 METHODS

### 2.1 Stocks used in the analysis and data collection

**FIGURE 2:**
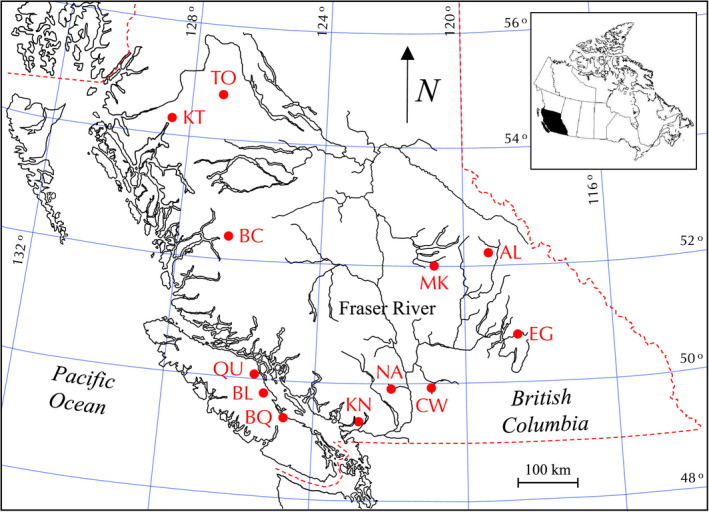
Locations where data were collected for environmental variables and life history traits of Coho Salmon across British Columbia. Abbreviations for spawning locations are as follows: Toboggan (TB), Kitimat (KT), McKinley (MK), Albreda (AL), Bella Coola (BC), Eagle (EG), Quinsam (QU), Black (BL), Coldwater (CW), Nahatlatch (NA), Big Qualicum (BQ), and Kanaka (KN)

Life history traits were compared for 12 populations of Coho Salmon selected from different regions across BC that represented a range of migration distances and potential incubation conditions experienced by this species (Figure 2). Data were obtained from Fisheries and Oceans Canada (DFO) and included annual mean female size (postorbital hypural length [*L*_POH_] most commonly or fork length [*L*_F_]), annual mean egg size (*M*_egg_), and annual mean fecundity (*F*) for each year data were available. Archived data were obtained from DFO facilities; Kanaka Creek Hatchery for the Kanaka Creek (KN) population, Big Qualicum Hatchery for the Big Qualicum River (BQ) population, Quinsam River Hatchery for the Quinsam River (QU) population, Snootli Creek Hatchery for the Bella Coola River (BC) population, Kitimat River Hatchery for the Kitimat River (KT) population, Spius Creek Hatchery for the Eagle River (EG) and Coldwater River (CW) populations, the Fraser River Stock Assessment Section (DFO) for data from Albreda Creek (AL) and the Nahatlatch River (NA) populations, Quesnel River Hatchery for the McKinley Creek (MK) population, and Toboggan Creek Hatchery for the Toboggan Creek (TB) population. Each population was assessed over multiple years, but data were not available for all life history traits for all years (Table 1). Additional life history data on mean *F* and *L*_POH_ were also obtained from Fleming and Gross (1990) for Big Qualicum River, Black Creek, and Quinsam River populations, and *L*_POH_ from Meldrum et al. (2017) and references therein for Black Creek.

**TABLE 1:** Summary of Coho Salmon life history trait data collected for each watershed. The range of years and the number of measurements for which mean values for female size at maturity as postorbital hypural length (*L*_POH_ [mm]), mean egg size (*M*_egg_ [mg]), mean fecundity (*F* [count]), and mean gonad size index (*I*_G_ [kg · m^‐3^]) available are listed. Sample sizes for measurements standardized to a common female spawner *L*_POH_ of 527 mm are provided in parentheses for *M*_egg_ and *F*. Sample sizes for models with absolute and standardized values for *I*_G_ were the same. Number of measurements for each life history trait used in the models are provided as a total

Watershed	Years	*L*_POH_	*M*_egg_	*F*	*I*_G_
Toboggan (TB)	1989–2008	20	20 [20]	20 [20]	20
Kitimat (KT)	1984–2013	14	14 [5]	30 [14]	5
McKinley (MK)	1984–2018	13	8 [1]	1 [1]	1
Albreda (AL)	2013–2018	6	–	–	–
Bella Coola (BC)	1999–2013	1	8 [–]	4 [–]	–
Eagle (EG)	1998–2013	8	8 [8]	8 [8]	8
Quinsam (QU)	1988–2014	6	6 [6]	5 [5]	5
Black (BL)	1984–2013	12	1 [1]	–	–
Coldwater (CW)	2000–2013	14	14 [14]	14 [14]	14
Nahatlatch (NA)	2000–2018	4	–	–	–
Big Qualicum (BQ)	1987–2014	10	9 [7]	13 [6]	5
Kanaka (KN)	2011–2014	4	5 [4]	5 [4]	4
Total		112	93 [66]	100 [72]	62

For female size, measurements for *L*_F_ were converted to *L*_POH_ according to the relationship established by Fleming and Gross (1990) and *L*_POH_ was used in all models; *L*_POH_ = 28.8 + *L*_F_ · 0.774. Egg size in mg (*M*_egg_) was used in all calculations, but only volume measurements were available for the Toboggan Creek population. Volume was transformed to mass by multiplying by the fraction of space occupied by random packed spheres (0.64; Finney, 1970) and the average density of salmon eggs (1.076; Bonham, 1976). A measure of reproductive investment, relative gonad size index (*I*_G_), was calculated from the average *M*_egg_ multiplied by average *F* and expressed as a ratio of gonad weight to *L*_POH_^3^ for each population and year that *M*_egg_, *F*, and *L*_POH_ were available.

Environmental variables were chosen based on their expected importance for explaining variation in *L*_POH_, *M*_egg_, *F*, and *I*_G_ for anadromous salmon. Environmental data were collected from global positioning system measurements of spawning locations, the Terrain Resource Information Management maps and databases of hatchery operations from DFO. The average latitude (LAT) for each population was used with an accuracy of ± 3 m; and was included because latitudinal clines in egg size, number, and reproductive investment have previously been documented (Fleming & Gross, 1990). Migration distance from the ocean to the spawning grounds (*D*_MIG_) was also calculated for each population as energy expenditure increases with migratory distance (Brett, 1995). Years of hatchery operations (*YOH*) was based on the difference in time between when DFO initiated supplemental stocking for a population and when the data used for the analysis were collected; *YOH* has been linked to decreases in egg size (Heath et al., 2003). We used stream width (*WD*) as a surrogate for river size which has been linked to productivity (Garcia de Leaniz et al., 2007); for our models, *WD* was categorized as either small (<5 m wetted width) or large (>5 m wetted width). Lakes limit temporal variation in dissolved organic matter, sediment loading, discharge, and daily temperature variation (Goodman et al., 2011; Milner & Bailey, 1989; Quinn, 2005); for our models, headwater (*HW*) sources were categorized as streams or lakes.

For each location, temperature loggers (HOBO U‐22; Onset Computer Corporation, Bourne MA) were placed in three different locations within the river mainstem or tributaries to the mainstem river (Tuor & Shrimpton, 2019). A surface temperature logger and two to three intragravel temperature loggers were deployed at each site within a study reach; intragravel loggers within a site were a minimum of three meters apart to capture some of the potential variation in temperatures from different redds within each system. Intragravel temperature loggers were buried at a depth of approximately 25 cm. Loggers recorded temperature hourly for approximately six months throughout incubation from November 2012 to May 2013. Additional temperature data were provided by DFO for the Quinsam River (2000 to 2017) and Big Qualicum River (2005 to 2018) watersheds. Mean surface water temperature was calculated during the peak of spawning from November 16 to 30 (*T*_NOV_); thermal experience during migration has been linked to energy expenditure and successful arrival to spawning grounds (Crossin et al., 2008; Wagner et al., 2005). Average intragravel temperature (*T*_AVG_) was calculated from November 16 to May 15 or until 750 ATU had been reached; thermal experience during incubation affects development (Johnston & McLay, 1997). Average intragravel temperature was calculated during the coldest period of incubation—the month of January (*T*_JAN_); incubation at cold temperature has been linked with increased mortality (Alderdice & Velsen, 1978; Murray et al., 1990; Tang et al., 1987). Northern interior populations of Coho Salmon in British Columbia experienced temperatures below 1°C early in embryo development less than a month after spawning and with fewer than 50 accumulated thermal units in some locations (Tuor & Shrimpton, 2019). Tolerance to low temperatures has been shown to increase with embryo age and mortality due to near‐freezing temperatures in laboratory studies may be dependent on stage of development (Alderdice & Velsen, 1978).

### 2.2 Statistical analysis

An information theoretic approach was used to evaluate competing models designed to explain the influence of environmental variables on the life history traits *L*_POH_, *M*_egg_, *F*, and *I*_G_. A positive relationship exists between *F* and female size (Braun et al., 2013; Campbell et al., 2006; Crone & Bond, 1976; Rounsefell, 1957) and larger females also produce larger eggs (Campbell et al., 2006; Heath et al., 2002; Heath et al., 2003; Kinnison et al., 1998, but see Drucker, 1972). The relationship between gonadal mass and female size is less clear as gonadal somatic index has been shown to be lower for larger females (Healey, 2001), greater for larger females (Campbell et al., 2006), or to have no pattern (Heath et al., 2002). We found a significant relationship between *M*_egg_ and *L*_POH_ (*F*_1,63_ = 48.4; *p* < .001), a significant relationship between *F* and *L*_POH_ (*F*_1,61_ = 97.2; *p* < .001), and also a significant relationship between *I*_G_ and *L*_POH_ (*F*_1,61_ = 6.94; *p* < .05). To determine whether female size influenced *M*_egg_, *F*, and *I*_G_, we constructed models for the absolute values of each measurement and also with measurements standardized to a common female spawner size of 527 mm (Beacham & Murray, 1993). Although our set of data included more measurements of *L*_POH_ (*n* = 122), than *M*_egg_ (*n* = 93), and *F* (*n* = 100), we were only able to obtain measurements of *L*_POH_ for some of the *M*_egg_ measurements (*n* = 66), and some of the *F* measurements (*n* = 72) for the different river systems and years. Consequently, we developed models with the larger set of data for *M*_egg_ and *F* and then developed models for the standardized *M*_egg[527]_ and *F*_[527]_ data with a subset of the data. As *L*_POH_ was used to calculate *I*_G_, sample size was the same for both model sets (*n* = 62).

The effects of environmental variables on life history traits were analyzed using truncated regression models to determine variation across each system. Truncated regression models were used to examine variation in life history traits as all values were positive. Variance inflation factors (VIF) were examined for each independent variable to determine if they resulted in multicollinearity within models. Variables with a VIF greater than 10 were considered to have a high degree of multicollinearity and not used in the model with their collinear variables. There was no evidence of collinearity between any of the variables (VIF < 10), except for the various measures of temperature. No models were constructed with more than one measure of temperature, rather top candidate models including the different measures of temperature were directly compared and ranked. Parameters for temperature were also tested to see whether use of a quadratic equation was appropriate based on comparison of the value of the small‐sample bias‐corrected Akaike's information criterion (AIC*_C_*) score with the same model with only the linear parameter as models of temperature effects are often improved using quadratic functions (Sykes et al., 2009). We fitted the annual mean values for each life history trait with multiple values per population. We clustered the data by river for each model as our observations for each population were not fully independent and life history traits for each river system were influenced by a common set of variables, but also to reduce potential weighting of the model by rivers with more years of data.

Akaike's information criterion, corrected for small sample sizes (AIC*_C_*), was used to rank models within candidate model sets (Burnham & Anderson, 2004). The model with the lowest AIC*_C_* in a candidate model set was accepted as the most parsimonious model, but those models within 2 AIC*_C_* units from the best approximating model were considered competitive (Burnham & Anderson, 2004). Akaike weights (*w_i_*), the relative likelihood that the candidate model was the best model given the data and the model set (Burnham & Anderson, 2004), are reported for each model. We interpreted parameter estimates from a single top model within the candidate model set when *w_i_* of the top model exceeded 0.90; in cases where there was model selection uncertainty (i.e., *w_i_* < 0.90), the model with ∆AIC*_C_* ≤ 2 and the fewest variables was selected as the preferred model (Burnham & Anderson, 2004). A jackknife approach was used to validate model performance two ways; by excluding one observation and constructing the model with the remaining observations and also by excluding one population and constructing the model with the remaining populations. Statistical analysis was performed using STATA statistical software (version IC 12; StataCorp, College Station, TX).

## 3 RESULTS

**FIGURE 3:**
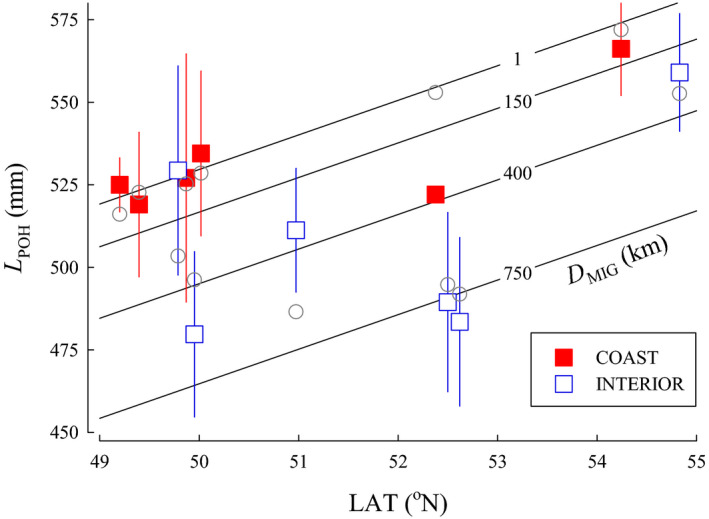
The relationship between postorbital hypural length (*L*_POH_), latitude (LAT), and migration distance from the ocean to spawning areas (*D*_MIG_) for populations of Coho Salmon from British Columbia. Isopleths for *D*_MIG_ were calculated from the model. Data are plotted as means ± 1 *SD* (squares). Model predictions for each population are plotted as circles

*L*_POH_—A total of 11 candidate models for Coho Salmon were included in the female size analysis; the top four models had a combined *w_i_* that added to approximately 0.9. For *L*_POH_ of female Coho Salmon at maturity, the best approximating model included LAT and *D*_MIG_ (Table 2). A second competitive model was similar, but included *T*_NOV_. The variables *T*_AVG_ and *T*_JAN_ were not incorporated in any of the candidate models for comparison. A third competitive model included LAT, *D*_MIG,_ and *WD*. The fourth model included *T*_NOV_, *D*_MIG_, *WD*, and *HW*. The top model containing LAT and *D*_MIG_ was the simplest and was accepted as the most parsimonious (Figure 3). For the top model, female *L*_POH_ increased with higher LAT (β = 10.48, SE = 1.62, 95% CI [7.31 to 13.6]) and decreased with *D*_MIG_ (β = −0.0867, SE = 0.0127, 95% CI [−0.112 to −0.0617]). Model parameters were still informative following jackknife validation as the confidence intervals did not overlap zero.

**TABLE 2:** Summary of Akaike's information criteria modified for small sample size (AIC*_C_*) ranking assessing the effect of environmental variables on Coho Salmon life history traits—postorbital hypural length (*L*_POH_) for female spawners, size of eggs (*M*_egg_), fecundity (*F*), and relative gonad size index (*I*_G_). To remove variation for *M*_egg_, *F* and *I*_G_ due to differences in female spawner size, models for these variables were also run with data standardized to a common *L*_POH_ of 527 mm as spawner size significantly affected these variables. Model parameters included latitude (LAT), average surface water temperature throughout the peak spawning period in November (*T*_NOV_), average intragravel water temperature throughout incubation from November to April (*T*_AVG_), migration distance for spawning locations within each river to the ocean (*D*_MIG_), stream width ranked as small or large (*WD*), total number of years an enhancement hatchery for Coho Salmon has been in operation within each watershed (*YOH*), and headwater source ranked as streams or lake (*HW*). The top four candidate models are shown. ΔAIC*_C_* is the difference in AIC*_C_* values between model and the best model of those considered, and *w_i_* is the probability that a model is the best model of the set

Variable	Model	K	AIC*_C_*	∆AIC*_C_*	*w_i_*
*L*_POH_	LAT + *D*_MIG_	3	1,049.2	0.0	0.598
*L*_POH_	LAT + *T*_NOV_ + *D*_MIG_	4	1,050.9	1.7	0.254
*L*_POH_	*T*_NOV_ + *D*_MIG_ + WD + *HW*	5	1,052.0	2.2	0.148
*L*_POH_	*T*_NOV_ + *D*_MIG_	3	1,081.8	32.8	0.000
*M*_egg_	*T*_AVG_ + *D*_MIG_ + WD + *HW*	5	737.5	0.0	0.724
*M*_egg_	*T*_AVG_ + *D*_MIG_ + WD + *YOH* + HW	6	739.9	2.1	0.252
*M*_egg_	LAT + *D*_MIG_ +WD + *YOH* + HW	6	745.0	7.6	0.017
*M*_egg_	LAT + *D*_MIG_ + WD + *HW*	5	746.5	9.1	0.008
*M*_egg[527]_	*T*_AVG_ + *D*_MIG_ + WD + *HW* + YOH	7	510.2	0.0	0.996
*M*_egg[527]_	*T*_AVG_ + *D*_MIG_ + WD + *HW*	6	522.0	11.8	0.003
*M*_egg[527]_	LAT + *D*_MIG_ + WD + *HW* + YOH	6	523.2	13.0	0.002
*M*_egg[527]_	LAT + *D*_MIG_ + WD + *HW*	5	528.6	18.4	0.000
*F*	LAT + *T*_NOV_ + WD + *HW*	5	1,494.5	0.0	0.982
*F*	LAT + *D*_MIG_	3	1504.6	10.1	0.006
*F*	LAT + *T*_NOV_ + WD	4	1506.0	11.5	0.003
*F*	LAT + *T*_NOV_	3	1506.1	11.6	0.003
*F*_[527]_	LAT + *T*_NOV_ +WD + *HW*	5	885.8	0.0	0.378
*F*_[527]_	LAT + *D*_MIG_	3	888.1	2.3	0.117
*F*_[527]_	LAT + *D*_MIG_ + *WD*	4	888.2	2.4	0.112
*F*_[527]_	LAT + *D*_MIG_ + *WD* + *HW*	5	888.8	3.0	0.083
*I*_G_	LAT + *T*_NOV_ + *D*_MIG_	4	75.4	0.0	0.414
*I*_G_	LAT + *D*_MIG_	3	77.4	2.0	0.152
*I*_G_	LAT + *T*_NOV_ + *D*_MIG_ + *WD*	5	78.0	2.2	0.138
*I*_G_	LAT+ + *D*_MIG_ + *WD* + HW	5	79.3	2.6	0.114
*I*_G[527]_	*D*_MIG_	2	80.0	0.0	0.302
*I*_G[527]_	LAT + *D*_MIG_	3	81.4	1.4	0.150
*I*_G[527]_	LAT + *T*_NOV_ + *D*_MIG_	4	81.6	1.6	0.136
*I*_G[527]_	LAT + *T*_NOV_	3	82.0	2.0	0.111

**FIGURE 4:**
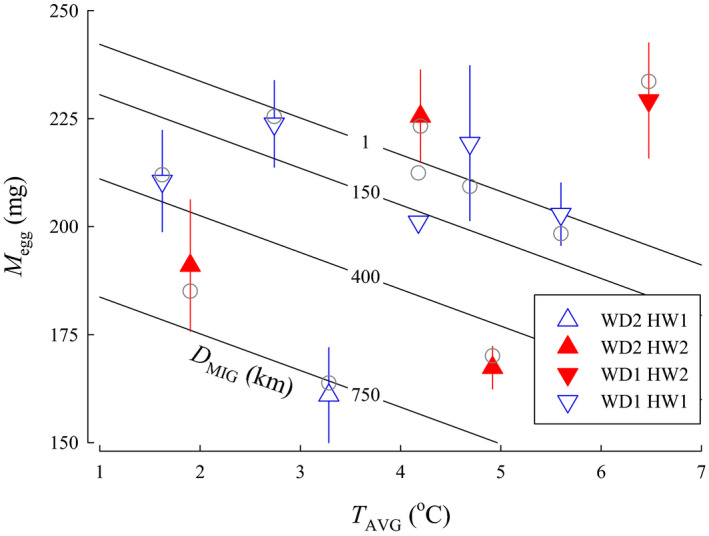
The relationship between egg size (*M*_egg_), average temperature for embryos and larvae during incubation (*T*_AVG_), and migration distance from the ocean to spawning areas (*D*_MIG_) for populations of Coho Salmon from British Columbia. Isopleths for *D*_MIG_ were calculated from the model for the categorical variables river width (*WD* = 1, small) and headwater source (*HW* = 1, stream). Data are plotted as means ± 1 *SD* (triangles) for *WD* small (1) or large (2) and *HW* stream (1) or lake (2). Model predictions for each population are plotted as circles

*M*_egg_—Models for egg size that included *T*_AVG_ performed better than models that included *T*_JAN_ or *T*_NOV_. The quadratic form of the model with *T*_AVG_ did not perform better. Consequently, candidate models that included temperature throughout incubation as a linear parameter (i.e., *T*_AVG_) were used for the analysis. A total of 12 candidate models for Coho Salmon were included in the *M*_egg_ analysis. The best approximating model for egg size variation showed *T*_AVG_, *D*_MIG_, *WD*, and *HW* were influential (Figure 4). The second‐ranked model was similar, but included *YOH*; however, *YOH* was uninformative as the confidence intervals overlapped zero. The top model, therefore, was selected as it was simpler, had an AIC*_C_* weight (*w_i_*) of 0.723, and ∆AIC*_C_* was > 2.0 between the top two models (Table 2). The top model indicated egg size increased with lower average incubation temperature (β = −8.51, *SE* = 1.17, 95% CI [−10.8 to −6.22]), shorter migration distance (β = −0.0780, *SE* = 0.0053, 95% CI [−0.0885 to −0.0676]), smaller size of the river system (β = −29.3, *SE* = 2.99, 95% CI [−35.1 to −23.4]), and in river systems with lakes as the headwater source (β = 38.5, *SE* = 4.16, 95% CI [30.4 to 46.7]). Model parameters were still informative following jackknife validation as the confidence intervals did not overlap with zero.

**FIGURE 5:**
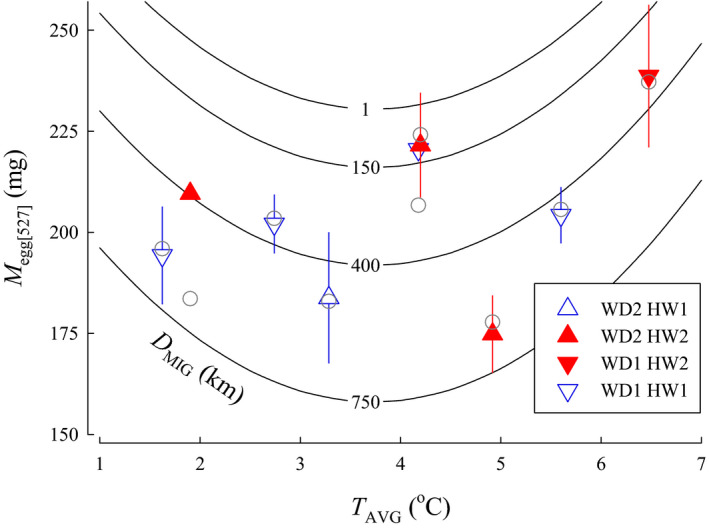
The relationship between egg size standardized to a common female spawner length of 527 mm (*M*_egg[527]_), average temperature for embryos and larvae during incubation (*T*_AVG_), and migration distance from the ocean to spawning areas (*D*_MIG_) for populations of Coho Salmon from British Columbia. Isopleths for *D*_MIG_ were calculated from the model for the categorical variables river width (*WD* = 1, small), headwater source (*HW* = 1, stream), and years of hatchery operation (YOH = 0). Data are plotted as means ± 1 *SD* (triangles) for *WD* small (1) or large (2) and *HW* stream (1) or lake (2). Model predictions for each population are plotted as circles

When *M*_egg_ was standardized to a common *L*_POH_ of 527 mm (*M*_egg[527]_), the ∆AIC*_C_* rankings were similar to the those for the *M*_egg_ models, except models with the quadratic equation for temperature performed much better. The top model for egg size variation had an AIC*_C_* weight (*w_i_*) of 0.996 and included *T*_AVG_, *D*_MIG_, *WD*, *HW*, and *YOH* (Table 2; Figure 5). For the top model, the linear component of the *T*_AVG_ quadratic was found to have a negative influence on *M*_egg[527]_ (β = −38.19, *SE* = 8.07, 95% CI [−54.0 to −22.3]), while the squared component of the quadratic had a positive influence (β = 5.12, *SE* = 1.09, 95% CI [2.98 to 7.26]). The model also indicated egg size increased with shorter migration distance (β = −0.0967, *SE* = 0.0140, 95% CI [−0.124 to −0.0692]), larger size of the river system (β = 21.4, *SE* = 8.23, 95% CI [5.28 to 37.5]), and in river systems with lakes as the headwater source (β = 11.9 *SE* = 3.59, 95% CI [4.86 to 18.9]). Egg size was also negatively associated with the years of hatchery operation (β = −0.524 *SE* = 0.450, 95% CI [−1.41 to 0.360]); however, *YOH* was uninformative. Model parameters were informative, except for *WD* and *HW*, following jackknife validation with exclusion of one observation as the confidence intervals did not overlap with zero. All model parameters were uninformative following jackknife validation with exclusion of one population and constructing the model with the remaining populations.

**FIGURE 6:**
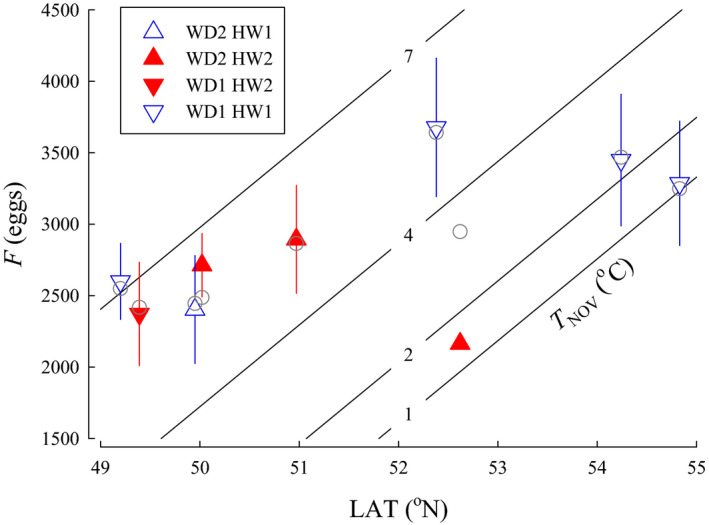
The relationship between fecundity (*F*), latitude (LAT), and average temperature in November when adults spawn (*T*_NOV_) for populations of Coho Salmon from British Columbia. Isopleths for *T*_NOV_ were calculated from the model for the categorical variables river width (*WD* = 1, small) and headwater source (*HW* = 1, stream). Data are plotted as means ± 1 *SD* (triangles) for *WD* small (1) or large (2) and *HW* stream (1) or lake (2). Model predictions for each population are plotted as circles

*F*—Models for fecundity that included *T*_NOV_ performed better than models that included *T*_AVG_ or *T*_JAN_. The quadratic form of the model with *T*_NOV_ did not perform better. Consequently, candidate models that included temperature as a linear variable during the spawning period were used for all subsequent analysis. A total of 11 candidate models were included in the fecundity analysis. The best approximating model for fecundity variation indicated that LAT, *T*_NOV_, *WD*, and *HW* were influential (Figure 6). The top model also had an AIC*_C_* weight (*w_i_*) greater than 0.9, indicating a clear top model (Table 2). For the top model, F increased with LAT (β = 572, *SE* = 31.3, 95% CI [511 to 633]), *T*_NOV_ (β = 418, *SE* = 30.6, 95% CI [358 to 478]), and *WD* (β = 1,203, *SE* = 113, 95% CI [982 to 1,425]), but decreased with *HW* (β = −1,085, *SE* = 114, 95% CI [−1,309 to −861]). Model parameters were still informative following jackknife validation as the confidence intervals did not overlap with zero.

**FIGURE 7:**
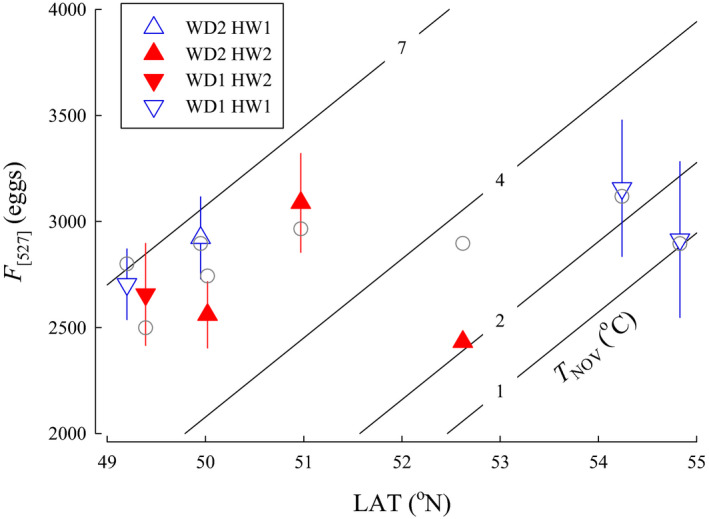
The relationship between fecundity standardized to a common female spawner length of 527 mm (*F*_[527]_), latitude (LAT), and average temperature in November when adults spawn (*T*_NOV_) for populations of Coho Salmon from British Columbia. Isopleths for *T*_NOV_ were calculated from the model for the categorical variables river width (*WD* = 1, small) and headwater source (*HW* = 1, stream). Data are plotted as means ± 1 *SD* (triangles) for *WD* small (1) or large (2) and *HW* stream (1) or lake (2). Model predictions for each population are plotted as circles

The top three models for *F*_[527]_ were the same as the top models for *F* (Table 2); models with the quadratic equation for temperature did not perform better. For the top model, *F* increased with LAT (β = 410, *SE* = 64.9, 95% CI [283 to 537]), *T*_NOV_ (β = 375, *SE* = 65.6, 95% CI [247 to 504]), and *WD* (β = 1,271, *SE* = 216, 95% CI [849 to 1695]), but decreased with *HW* (β = −1,140, *SE* = 215, 95% CI [−1,560 to −719]) (Figure 7). Model parameters were still informative following jackknife validation with exclusion of one observation, but all parameters were uninformative following jackknife validation with exclusion of one population.

**FIGURE 8:**
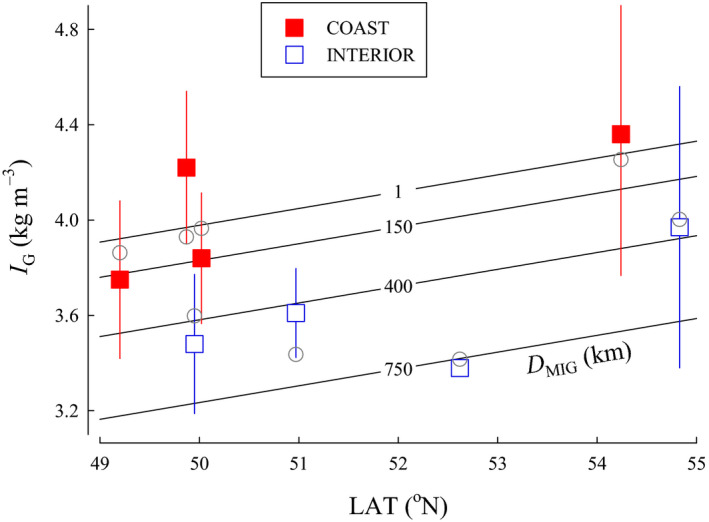
The relationship between relative gonad size index (*I*_G_), latitude (LAT), and migration distance from the ocean to spawning areas (*D*_MIG_) for populations of Coho Salmon from British Columbia. Isopleths for *D*_MIG_ were calculated from the model. Data are plotted as means ± 1 *SD* (squares). Model predictions for each population are plotted as circles

*I*_G_—A total of 11 candidate models for Coho Salmon were included in the relative gonad size index (*I*_G_) analysis. The top model for *I*_G_ included LAT, *T*_NOV_, and *D*_MIG_. Models with *T*_AVG_ or *T*_JAN_ were not examined. The quadratic form of the model with *T*_NOV_ did not perform better. A second competitive model was similar, but did not include *T*_NOV_. The second‐ranked model included LAT and *D*_MIG_ had a ∆AIC*_C_* ≤ 2 from the model ranked first and, because of the marginal difference in ∆AIC*_C_* scores, we determined the best model to be the one with the fewest variables (Table 2). The selected model indicated that *I*_G_ increased with LAT (β = 0.0706, *SE* = 0.0183, 95% CI [0.0347 to 0.106]), but was lower with longer *D*_MIG_ (β = −0.000994, *SE* = 0.000284, 95% CI [−0.00155 to −0.000438]; Figure 8). Model parameters were still informative following jackknife validation when a single value was excluded, but became uninformative when populations were excluded as the confidence intervals overlapped zero.

**FIGURE 9:**
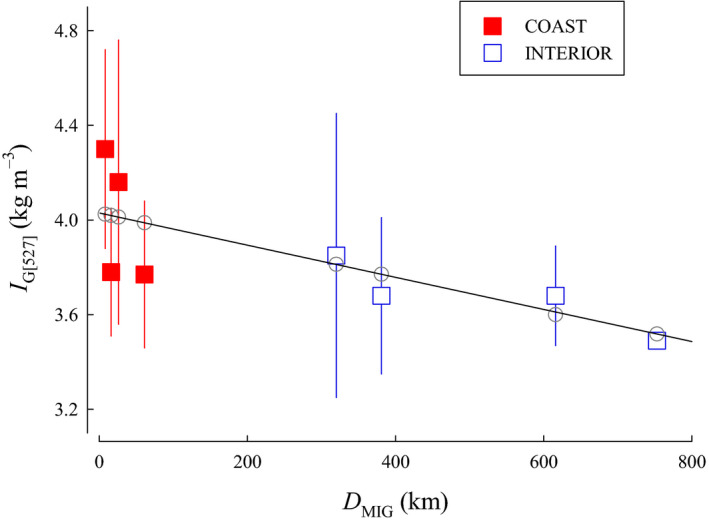
The relationship between relative gonad size index standardized to a common female spawner length of 527 mm (*I*_G[527]_) and migration distance from the ocean to spawning areas (*D*_MIG_) for populations of Coho Salmon from British Columbia. Data are plotted as means ± 1 *SD* (squares). Model predictions for each population are plotted as circles

The top model for *I*_G[527]_ included *D*_MIG_, but only had an AIC*_C_* weight (*w_i_*) of 0.302 (Table 2). Other competitive models had more variables and greater AIC*_C_* values. Consequently, we determined the best model to be the one with the fewest variables. Top model indicated that *I*_G_ decreased with longer *D*_MIG_ (β = −0.000679, *SE* = 0.000261, 95% CI [−0.00119 to −0.000167]; Figure 9). *D*_MIG_ was informative following jackknife validation when a single value was excluded, but uninformative when populations were excluded.

## 4 DISCUSSION

Our study furthers our understanding of spatial and environmental factors that affect life history variation for a species with a wide geographic distribution, but also includes variables of importance that have seldom been included in models developed across a wide spatial scale—migration distance and temperature. A number of previous studies have examined life history variation among populations at different latitudes, but the populations have been limited to coastal watersheds or short migration distances into interior river systems (Fleming & Gross, 1989, 1990; Beacham, 1982; but see Beacham & Murray, 1993). In our study, data were collected from populations of Coho Salmon that migrate <20 km to stocks that spawn more than 750 km from the ocean, in rivers that range in latitude from 49 to 55 °N, and that differ in river size and headwater source.

Fleming and Gross (1989) reported that both body depth and length of female Coho Salmon decreased with increased migration distances, although the maximum freshwater migration distance in their study was only 150 km. Taylor and McPhail (1985) found that interior Fraser juvenile Coho Salmon have a more fusiform (streamlined) body shape in comparison with costal populations, a pattern that may persist throughout life into adult migration. Adults from interior populations with more energetically demanding migrations may also have distinctive fusiform morphology as it is superior for sustained swimming compared with costal populations having more robust bodies (Fleming & Gross, 1989; Taylor & McPhail, 1985).

Female size and morphology have previously been shown to be influenced by total reproductive investment (higher in larger females), migration distance (thinner, smaller fish with longer caudle peduncle in streams with longer migration distance), and competition for redd location on spawning grounds (more pronounced kype, larger body size, and brighter colouration with increased competition) (Fleming & Gross, 1989). A number of studies have shown that larger females produce more large eggs than smaller females—an overall increase in reproductive investment (Beacham & Murray, 1993; Braun et al., 2013; Rounsefell, 1957). van den Berghe and Gross (1989) found female size contributed to overall fitness in three ways, increase initial biomass of egg production, the ability to acquire a high‐quality territory for redds, and success in nest defense. Thus, in smaller systems that may have limited spawning habitat and higher competition, larger females are expected to be more successful—yet we found no effect of stream size on *L*_POH_. Although head water type (stream vs. lake) is expected to influence the productivity and temperature of the downstream river (Garcia de Leaniz et al., 2007) and females should be larger in lake fed systems, system type was also not in the top models.

In our model, egg size increased for river systems with colder *T*_AVG_ during incubation for the model using absolute egg size—a relationship that has not been previously demonstrated. In fact, the opposite trend was expected as earlier studies found egg size in Coho Salmon decreased in more northern populations (Beacham & Murray, 1993; Fleming & Gross, 1990), which are watersheds with colder incubation temperatures (Tuor & Shrimpton, 2019). Models using egg size data standardized to a common *L*_POH_ of 527 mm were improved, however, with a quadratic equation for *T*_AVG_ and the squared term was positive indicating that at the warmest temperatures egg sized increased. Our model suggested that *M*_egg[527]_ was minimized for incubation temperatures of approximately 3.5°C. Earlier work suggested that lower water temperature would lower metabolic rate and increase yolk conversion efficiency (Johnston & McLay, 1997; Killeen et al., 1999; Stickland et al., 1998), allowing reduced egg provisioning without a subsequent loss in growth potential (Johnston & McLay, 1997; Stickland et al., 1998). Murray et al. (1990) also found that northern and mainland stocks incubated at low temperatures (1.5 to 2.0°C) produced larger, heavier alevin and fry than southern and coastal populations—suggesting potential genetic differences among populations. The lowest temperatures examined in the studies cited above were between 1.5 and 2.0°C, warmest temperatures examined up to 15°C, and over this range of temperature metabolic efficiencies may favor smaller eggs at lower temperatures. Temperatures for some of the populations of Coho Salmon examined in our current study, however, were colder than the experimental conditions reported for these studies. Average January temperatures within redds were 0.34°C and 0.70°C for spawning locations in McKinley Creek and Toboggan Creek, respectively (Tuor & Shrimpton, 2019). Additionally, temperatures below 1°C occurred early in development (Tuor & Shrimpton, 2019)—at a stage that previous laboratory studies have shown resulted in poor survival or complete mortality (Alderdice & Velsen, 1978; Tang et al., 1987). Thus, when temperatures reach near‐freezing, the fitness relationship between water temperature and egg size may reverse. Metabolic rates of fish incubating at near‐freezing temperatures are not well understood, but development at such cold temperatures may be metabolically costly—necessitating larger eggs.

Although LAT was in two of the top four models for egg size, it was not in the top ranked model which in addition to *T*_AVG_, included *D*_MIG_, *WD*, *HW*, and *YOH* for *M*_egg[527]_ indicating a complex relationship between rearing environment and the evolution of egg size. The geographic distribution in the study by Fleming and Gross (1990) did not extend as far north as our study, and the cold temperatures experienced by their populations would not have been as extreme as our study. Longer migrations and more energetically demanding migrations have been linked to a decrease in egg size (Kinnison et al., 2001). Distance of freshwater migration to the spawning grounds is generally reported to have a marked effect on egg size, with populations of Sockeye Salmon, Chinook Salmon, and Coho Salmon spawning in the upper portions of the drainages in large rivers (such as the Fraser River in British Columbia) having reduced egg size compared with coastal spawning populations (Beacham & Murray, 1993). Smaller eggs with an increase in *D*_MIG_ have been linked to energetic cost. For upper Fraser River Sockeye Salmon that migrate approximately 1,100 km to spawning areas, in years with more difficult migrations due to higher water discharge, females invested less in gonads and produced smaller, but not fewer, eggs (Braun et al., 2013).

For females spawning in smaller river systems, the absolute size of the eggs was larger on average. Bedload movement is driven by stream size, gradient, and hydrologic regimes, consequently smaller rivers are often characterized by smaller substrate due to lower flow that will not wash away finer material (Knighton, 1998). Smaller eggs with greater surface‐area to volume are expected in systems with smaller gravel size due to reduced porosity and oxygen levels (Quinn et al., 1995; Rollinson & Hutchings, 2011). Our model using *M*_egg[527]_, however, found that standardized egg size was larger for larger river systems. Einum et al. (2002) found that larger eggs survived better at low dissolved oxygen levels than small eggs—suggesting that substrate size may not constrain egg size. Additionally, McRae et al. (2012) showed that Coho Salmon spawn in locations with significantly higher hyporheic dissolved oxygen levels, but with no difference in gravel size. Consequently, other factor(s) may be selecting for egg size.

Eggs were also larger in rivers with headwater lakes, rivers that tend to have greater hydrograph stability. Channel bed stability was found to be greater in reaches of streams downstream of lakes than in stream reaches with no upstream lakes (Milner et al. 2000). The greater hydrographic stability in rivers below lakes, therefore, may allow larger eggs to survive in more stable channel beds. Generally larger rivers (Nahatlatch River and Eagle River) and systems with lakes in the headwaters (Nahatlatch and Eagle Rivers, but not McKinley Creek) also tended to be more thermally stable (Tuor & Shrimpton, 2019). Fluctuations in temperature result in reduced growth compared with constant temperature even when the accumulated thermal units are the same (Meeuwig et al., 2004; Shrimpton et al., 2007). Reduced growth may be due to increased metabolic rate associated with oscillating temperature regimes. Standard metabolic rate was more than 25% higher for Atlantic salmon parr maintained under a fluctuating thermal regime (greater than ± 2.0°C) than salmon held at a relatively constant water temperature (± 0.5°C; Beauregard et al., 2013). Consequently, river systems with more variable thermal regimes of the intragravel environment may select for larger eggs to benefit growth and development in developing larval Coho Salmon.

The number of years of hatchery enhancement within a system was previously shown to have a positive effect on egg size. Fleming and Gross (1990) and Quinn et al. (2004) found Coho Salmon eggs were larger for hatchery populations than wild populations. The data of Fleming and Gross (1990), however, were later reanalyzed by Beacham and Murray (1993); addition of a regional variable resulted in a lack of difference among populations. In contrast, Heath et al. (2003) and Haring et al. (2016) found that greater *YOH* resulted in smaller eggs; enhancement programs relaxed selective pressure for larger eggs in Chinook salmon, driving selection for decreased gonadal investment, increased fecundity, and smaller eggs. Although, *YOH* was in the top model for *M*_egg[527]_ and suggested a negative relationship between egg size and hatchery supplementation, it was uninformative in our analysis.

The relationship we observed between absolute *F* and LAT could be accounted for by larger fish observed at higher LAT. An increase in fecundity with latitude has been suggested to be a reproductive trade‐off to produce more, smaller eggs rather than few, large eggs (Beacham, 1982; Beacham & Murray, 1993; Fleming & Gross, 1990). Analysis of absolute *F* and *M*_egg_ suggested that a reproductive trade‐off was not found among the Coho Salmon populations in our study. The positive relationship between *F*_[527]_ and LAT indicates that females are also relatively more fecund at higher latitude, but *M*_egg[527]_ showed a complex pattern. Standardized egg size was larger for populations from watersheds with the coldest *T*_AVG_ which tended to be the most northern and interior populations. Although LAT was not in the top model for *I*_G[527]_, LAT was in the next 3 models and found to be positive and influential suggesting that a reproductive trade‐off did not exist in our study. Increased net reproductive investment in the more northern populations, such as reported here, may reflect better adult ocean foraging opportunities, balanced by lower offspring survival likelihood.

Temperature during the spawning period, *T*_NOV_, had a positive influence on absolute and relative fecundity such that females who spawned in warmer water temperatures were more fecund. Systems with higher temperatures for a given latitude were found along the coast in warmer, wetter climates, with longer growth seasons. The model also showed that females spawning in larger rivers had higher fecundity; these rivers were found mostly within the cooler interior watersheds. The positive effect of *T*_NOV_ and *WD* on *F*, however, may be influenced by *M*_egg_ which was inversely related to *T*_AVG_ and *WD*. Similarly, the negative relationship between *HW* and *F*, may be influenced by the positive relationship between *HW* and *M*_egg_.

Surprisingly, we did not find an effect of *D*_MIG_ on *F*. Freshwater migration distance to spawning ground had a negative effect on fecundity in Pacific salmon (Beacham & Murray, 1993). Similarly, Manzer and Miki (1985) estimated that coastal populations of sockeye salmon were approximately 18% more fecund that interior stocks in British Columbia. In contrast, Kinnison et al. (2001) found a positive relationship between migration distance and *F*, but these authors also showed that populations that migrated farther invested more in ovarian mass with *F* more than *M*_egg_ accounting for the difference. Difficulty of migration to the spawning grounds also affects morphology and life history traits. Salmon are more streamlined with increasing migration difficulty (Fleming & Gross, 1989), and the biomass of eggs produced declines (Fleming & Gross, 1989; Figure 9). Longer and more difficult migrations should reduce the amount of energy available for egg production resulting in either smaller eggs (Braun et al., 2013; Crossin et al., 2004; Kinnison et al., 2001) or a reduction in the number of eggs produced (Fleming & Gross, 1989). Variation in *M*_egg_ with migration difficulty (or *D*_MIG_) would suggest trade‐offs and constraints favoring offspring number over offspring size. Migration, therefore, may strongly influence patterns of reproductive allocation, favoring egg number over egg size (Kinnison et al., 2001). Additionally, Braun et al. (2013) found that in years when sockeye salmon upstream migration was more energetically challenging due to high water discharge, females reached spawning streams with smaller gonads by producing smaller eggs, but the number of eggs produced did not change—suggesting that egg size may be plastic quite late in maturation, but egg number may be set earlier.

Although, *I*_G_ varied with LAT and *D*_MIG_, how gonadal mass was partitioned gives insight into the trade‐off between *M*_egg_ and *F* that will maximize fitness (Smith & Fretwell, 1974). We examined *M*_egg_ and *F* for Coho Salmon populations that inhabit a wide range of environmental conditions over multiple years and both variables varied among the populations examined, but variation in *F* among years was greater than *M*_egg_ among years. The coefficient of variation (CV) among years for populations where we had multiple years of data for both *F* and *M*_egg_ were consistent among populations, but CV was approximately 3‐fold greater for *F* than *M*_egg_; 0.127 ± 0.026 and 0.048 ± 0.011 for *F* and *M*_egg_, respectively. Fleming and Gross (1990) also found significant variation in *F* among years, whereas *M*_egg_ was more consistent among years. Thus, *M*_egg_ appears to be more canalized for populations we examined, while *F* may vary in response to the total amount of energy available. Local optima for egg size may drive the positive relationship between egg number and latitude—a pattern that is partially off‐set by larger female size and relative gonad size index with latitude. Individual *M*_egg_, however, is likely to be more plastic following initiation of maturation as *F* did not change during upriver migration (Braun et al., 2013); a reduction in provisioning to eggs due to greater energetic requirement will result in smaller *M*_egg_. Ultimately, timing of the selective pressure will contribute to differences that are observed in the phenotype.

We found that latitude of spawning grounds, migratory distance, temperature, and hydrologic features were influential in shaping patterns of reproductive investment in female Coho Salmon. Trade‐offs existed in how gonadal mass was partitioned, but some relationships were not as expected or opposite to our expectation. Both intensity and direction of natural selection may change in environments that vary temporally, which in turn would favor generalist genotypes or phenotypic plasticity over local adaptation (Fraser et al., 2011). Observed phenotypes, therefore, may vary dependent on constraints that occur at different life history stages and account for the temporal variation in phenotype we observed among populations.

## CONFLICT OF INTEREST

None declared.

## AUTHOR CONTRIBUTIONS

**Kimberly M. F. Tuor:** Data curation (lead); Formal analysis (lead); Investigation (lead); Writing‐original draft (lead). **Daniel D. Heath:** Conceptualization (equal); Supervision (supporting); Writing‐review & editing (supporting). **J. Mark Shrimpton:** Conceptualization (equal); Formal analysis (supporting); Funding acquisition (lead); Investigation (supporting); Project administration (lead); Supervision (lead); Writing‐review & editing (lead).

## ETHICAL APPROVAL

Permission for sampling was granted by the University of Northern British Columbia UNBC Animal Care and Use Committee (protocols 2012–04; 2013–19).

## Data Availability

Biological and physical data files: Tuor, Kim; Heath, Daniel; Shrimpton, Mark (2020), Dataset for: Spatial and environmental effects on Coho Salmon life history trait variation, Dryad, Dataset, https://doi.org/10.5061/dryad.nk98sf7qq
